# Omega-3 Polyunsaturated Fatty Acids Decrease Aortic Valve Disease Through the Resolvin E1 and ChemR23 Axis

**DOI:** 10.1161/CIRCULATIONAHA.119.041868

**Published:** 2020-06-08

**Authors:** Gonzalo Artiach, Miguel Carracedo, Oscar Plunde, Craig E. Wheelock, Silke Thul, Peter Sjövall, Anders Franco-Cereceda, Andres Laguna-Fernandez, Hildur Arnardottir, Magnus Bäck

**Affiliations:** 1 Department of Medicine (G.A., M.C., O.P., S.T., A.L.-F., H.A., M.B.), Karolinska Institutet, Stockholm, Sweden.; 2 Division of Physiological Chemistry II, Department of Medical Biochemistry and Biophysics, (C.E.W.), Karolinska Institutet, Stockholm, Sweden.; 3 Department of Molecular Medicine and Surgery(A.F.-C.), Karolinska Institutet, Stockholm, Sweden.; 4 Chemistry, Biomaterials and Textiles, RISE Research Institutes of Sweden, Borås, Sweden (P.S.).; 5 Theme Heart and Vessels, Division of Valvular and Coronary Disease, Karolinska University Hospital, Stockholm, Sweden. (A.F.-C., M.B.).

**Keywords:** calcification, physiologic, fatty acids, omega-3, heart valve diseases, inflammation, lipids

## Abstract

**Background:**

Aortic valve stenosis (AVS), which is the most common valvular heart disease, causes a progressive narrowing of the aortic valve as a consequence of thickening and calcification of the aortic valve leaflets. The beneficial effects of omega-3 polyunsaturated fatty acids (n-3 PUFAs) in cardiovascular prevention have recently been demonstrated in a large randomized, controlled trial. In addition, n-3 PUFAs serve as the substrate for the synthesis of specialized proresolving mediators, which are known by their potent beneficial anti-inflammatory, proresolving, and tissue-modifying properties in cardiovascular disease. However, the effects of n-3 PUFA and specialized proresolving mediators on AVS have not yet been determined. The aim of this study was to identify the role of n-3 PUFA–derived specialized proresolving mediators in relation to the development of AVS.

**Methods:**

Lipidomic and transcriptomic analyses were performed in human tricuspid aortic valves. Apoe^−/−^ mice and wire injury in C57BL/6J mice were used as models for mechanistic studies.

**Results:**

We found that n-3 PUFA incorporation into human stenotic aortic valves was higher in noncalcified regions compared with calcified regions. Liquid chromatography tandem mass spectrometry–based lipid mediator lipidomics identified that the n-3 PUFA–derived specialized proresolving mediator resolvin E1 was dysregulated in calcified regions and acted as a calcification inhibitor. Apoe^−/−^ mice expressing the *Caenorhabditis elegans* Fat-1 transgene (Fat-1^tg^×Apoe^−/−^), which enables the endogenous synthesis of n-3 PUFA and increased valvular n-3 PUFA content, exhibited reduced valve calcification, lower aortic valve leaflet area, increased M2 macrophage polarization, and improved echocardiographic parameters. Finally, abrogation of the resolvin E1 receptor ChemR23 enhanced disease progression, and the beneficial effects of Fat-1^tg^ were abolished in the absence of ChemR23.

**Conclusions:**

n-3 PUFA-derived resolvin E1 and its receptor ChemR23 emerge as a key axis in the inhibition of AVS progression and may represent a novel potential therapeutic opportunity to be evaluated in patients with AVS.

## Abstract 2

Supplemental Digital Content is available in the text.

**Clinical Perspective**

**What Is New?**

- Omega-3 polyunsaturated fatty acids (n-3 PUFAs) have not previously been associated with valvular heart disease.
- This study shows that human stenotic aortic valves contained decreased levels of n-3 PUFAs and that n-3 PUFA treatment decreased aortic valve calcification and aortic valve leaflet area in murine models, concomitant with improved aortic valve hemodynamics.
- The proresolving lipid mediator resolvin E1, which is derived from the n-3 PUFA eicosapentaenoic acid, exerted protective effects on valvular interstitial cell calcification and valvular inflammation though its receptor ChemR23.

**What Are the Clinical Implications?**

- The n-3 PUFA/resolvin E1/ChemR23 axis emerges as a protective pathway in aortic stenosis.
- Clinical evaluation of n-3 PUFA treatment may open up novel therapeutic opportunities for preventing the progression of aortic valve stenosis.

Aortic valve stenosis (AVS) is the most common valvular heart disease and major cause of heart failure and increased cardiovascular mortality. When severe, AVS causes significant cardiac outflow obstruction with 1-year mortality rate reaching 60% for severe symptomatic AVS.^1^ The progressive aortic valve narrowing develops as a consequence of an increased thickening and calcification of the aortic valve leaflets.^2^ A vast majority of clinical trials has failed to stop the progression of AVS,^3^ and for that reason, the only available therapeutic treatment relies on aortic valve prosthesis implantation.^4^

A recent clinical trial demonstrated that eicosapentaenoic acid (EPA) ethyl ester in high doses conferred a 25% relative risk reduction in major cardiovascular events compared with placebo, including cardiovascular death, nonfatal myocardial infarction, nonfatal stroke, coronary revascularization, and hospitalization for unstable angina as outcome.^5^ Omega-3 polyunsaturated fatty acids (PUFAs), especially EPA and docosahexaenoic acid (DHA), are known for their anti-inflammatory and proresolving properties by serving as substrates for the biosynthesis of a unique class of bioactive lipid mediators called specialized proresolving lipid mediators (SPMs).^6^

The SPM-derived from EPA and DHA include the E-series and D-series resolvins, protectins, and maresins. Under certain conditions, EPA- and DHA-derived SPMs are actively formed by a wide variety of cells and are responsible for the resolution of the inflammatory process.^7^ Resolvins mediate their signaling through the specific G-protein–coupled receptors, denoted ChemR23, GPR18, ALX/FPR2, and GPR32.^8^ Indeed, beneficial effects of resolvin E1 (RvE1) through ChemR23 signaling have been demonstrated in the context of atherosclerosis,^9^ intimal hyperplasia,^10^ and vascular calcification.^11^ However, the role of n-3 PUFAs, specifically the effect of RvE1 through ChemR23 in the context of AVS and valve calcification and inflammation, remains unknown.

The aim of this study was to identify the role of n-3 PUFA–derived SPMs in relation to the development of AVS. In human stenotic valves, we identified the n-3 PUFA–derived SPMs RvE1 and its receptor ChemR23, and we studied the valvular effects in 2 different mouse models, demonstrating the beneficial effects mediated through the RvE1/ChemR23 axis.

## Methods

### Patients

Tricuspid human aortic valves were obtained from 96 patients undergoing aortic valve replacement surgery. The study was approved by the local ethics committee (2012/1633) and was in agreement with the Declaration of Helsinki. All patients gave informed consent.

### Liquid Chromatography–Tandem Mass Spectrometry

Valve samples were placed in ice-cold methanol containing deuterated internal standards representing each chromatographic region of identified lipid mediators (500 pg each) before processing to facilitate quantification and assessment of sample recovery. Samples were homogenized and proteins were precipitated, followed by solid-phase extraction with Isolute C18 columns (Biotage, Uppsala, Sweden)^12^ and targeted liquid chromatography–tandem mass spectrometry as previously described.^13,14^ The system consisted of Waters XevoTQS triple quadruple equipped with Acquity UPLC System from Waters Corporation and an autosampler cooled to 5°C (Milford, MA). An Acquity UPLC BEH (Ethylene Bridged Hybrid) C18 column (130 Å, 1.7 μm, 2.1×150 mm) equipped with a precolumn (Acquity UPLC C18 VanGuard Pre-Column, 130 Å, 1.7 μm, 2.1×5 mm; Milford, MA) was used with gradients A (0.1% acetic acid in water) and B (acetonitrile/isopropanol; 90:10 [vol/vol−1]) from 80:20 (vol/vol−1) to 0:100 (vol/vol−1) in 17 minutes at a flow rate of 0.5 mL·min^−1^, which was then equilibrated to initial conditions for 2.5 minutes. To monitor and quantify levels of lipid mediators, a multiple reaction monitoring method was developed with a signature ion pairs Q1 (parent ion) to Q3 (characteristic daughter ion) for each molecule (eg, Q1>Q3 for RvE1 349>195; resolvin D3 (RvD3), 375>147; and leukotriene B4, 339>195). Linear calibration curves were obtained using synthetic standards for each lipid mediator, which gave *R*^2^ values of 0.98 to 0.99. Data acquisition was performed in the negative ionization mode, and identification was conducted in accordance with published criteria.^14^

### Animals

Animals were bred and kept under standard housing conditions, and all animal experiments were conducted in accordance with guidelines from Directive 2010/63/EU of the European Parliament on the protection of animals used for scientific purposes and were approved by the Ethical Committee of Northern Stockholm (Ethical Permit N28/15).

ChemR23^−/−^ mice were purchased from Deltagen. Male mice were used in all experiments. Double-knockout Apoe^−/−^×ChemR23^−/−^ were generated by crossbreeding ChemR23^−/−^ mice on a C57BL/6J background with apolipoprotein E–deficient (Apoe^−/−^) mice also on C57BL/6J background. C57BL/6J mice hemizygous for the transgene *Caenorhabditis elegans* Fat-1^tg^ were a gift from Professor Joan Clària (Barcelona, Spain). The Fat-1^tg^ encodes a desaturase that converts n-6 to n-3 PUFA.^15^ Fat-1^tg^×Apoe^−/−^×ChemR23^+/+^ and Fat-1^tg^×Apoe^−/−^×ChemR23^−/−^ mice were generated by crossbreeding Fat-1 transgenic mice with the Apoe^−/−^ strains described above.

### Statistics

Results are expressed as mean±SEM. Statistical significance of differences for normally distributed data were assessed with paired or unpaired Student *t* test when comparing 2 groups and with 1-way or 2-way ANOVA as appropriate followed by a Holm-Sidak test for multiple comparisons. Repeated measures analysis was performed when appropriate and possible. Mixed-effects ANOVA was applied when accounting for technical replicates. Partial *R*^2^ was established for the associations within and between morphological and echocardiographic parameters. In brief, partial *R*^2^ was calculated for the quantitative predictor as the percent reduction in the error sum of squares between 2 models: the first model including genotype as a predictor and the second model including the quantitative predictor.^16^ For categorical data, statistical significance was assessed with the Fisher exact test. Statistical significance was assigned at *P*<0.05. Statistical analyses were performed with GraphPad Prism 8 (GraphPad Software Inc, La Jolla, CA) and NCSS version 9 (LLC, East Kaysville, UT).

Details about experimental animals (Tables IV through VII and IX and Figures I and II in the Data Supplement), human data (Tables I, II, and VIII in the Data Supplement), materials (Table III in the Data Supplement), and supplemental methods are provided online in the Data Supplement. One author had full access to all the data in the study and takes responsibility for its integrity and the data analysis. Data, methods, and materials will be available to other researchers for the purposes of reproducing the results or replicating the procedures (available at the authors’ laboratories).

## Results

### n-3 PUFA Content in Human Aortic Valves

**Figure 1.**
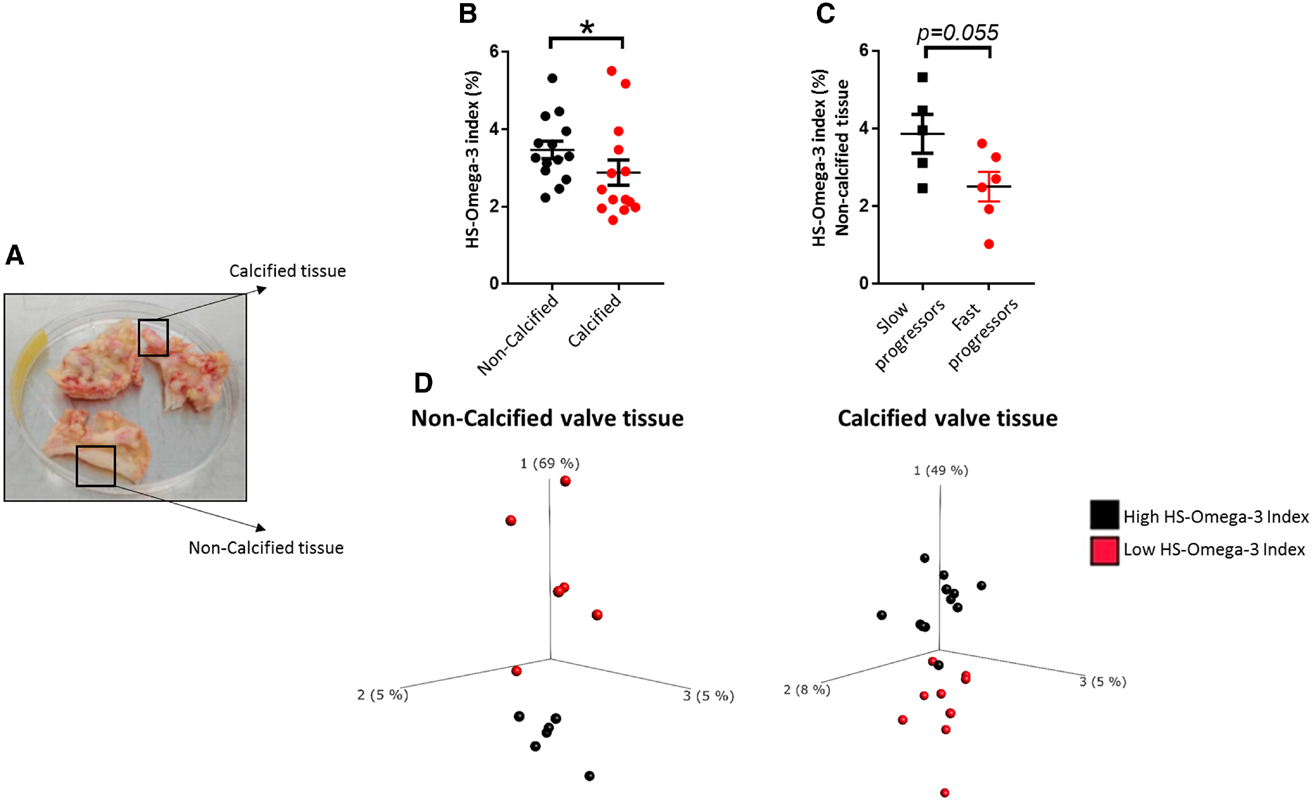
**HS-omega-3 index is decreased in calcified aortic valve tissue and is lower in noncalcified valve tissue of aortic valve stenosis (AVS) fast progressors.** **A**, Aortic valves from patients undergoing aortic valve replacement were dissected into noncalcified and calcified valve tissue. **B**, HS-omega-3 index measured by gas chromatography in calcified and noncalcified valve tissue from the same patients (n=14). **C**, HS-omega-3 index in patients with AVS with slow and fast progression in noncalcified (n=5 and n=6, respectively) valve tissue. Data are presented as individual values; horizontal lines represent mean±SEM. Statistical significance was evaluated using either paired (**B**) or unpaired (**C**) Student *t* test. *P<0.05. **D**, Transcriptomics-based principal component (PC) analysis of noncalcified (n=6 high, n=6 low HS-omega-3 index) and calcified (n=12 high, n=9 low HS-omega-3 index) human valve tissue. The 3 axes represent the principal components displaying maximum variability between data sets, in which PC1 represents the axis with most variability.

The fatty acid content in human aortic valves, as determined by gas chromatography analysis of noncalcified aortic valve tissue derived from n=21 patients, is shown in the Table. Comparing noncalcified and calcified tissue derived from the same aortic valve (Figure 1A) revealed that the HS-omega-3 index, an overall measure of the n-3 PUFA content, was significantly higher in noncalcified parts compared with calcified parts of the aortic valve (Figure 1B). There were no significant differences between the characteristics of patients with aortic valve HS-omega-3 index above and below median (Table I in the Data Supplement). However, patients who had a history of slow AVS progression exhibited a higher HS-omega-3 in noncalcified aortic valve regions compared with patients with rapidly progressing AVS (Figure 1C), although the limited number of observations did not attain statistical significance (*P*=0.055). In addition, principal component analysis revealed differential transcriptomic profiles between patients with high and low HS-omega-3 index in the noncalcified and also calcified human aortic valve tissue (Figure 1D). The underlying GSEA KEGG pathways differentiating high and low HS-omega-3 index in noncalcified and calcified human aortic valve tissue, respectively, are shown in Table II in the Data Supplement.

**Table 1.**
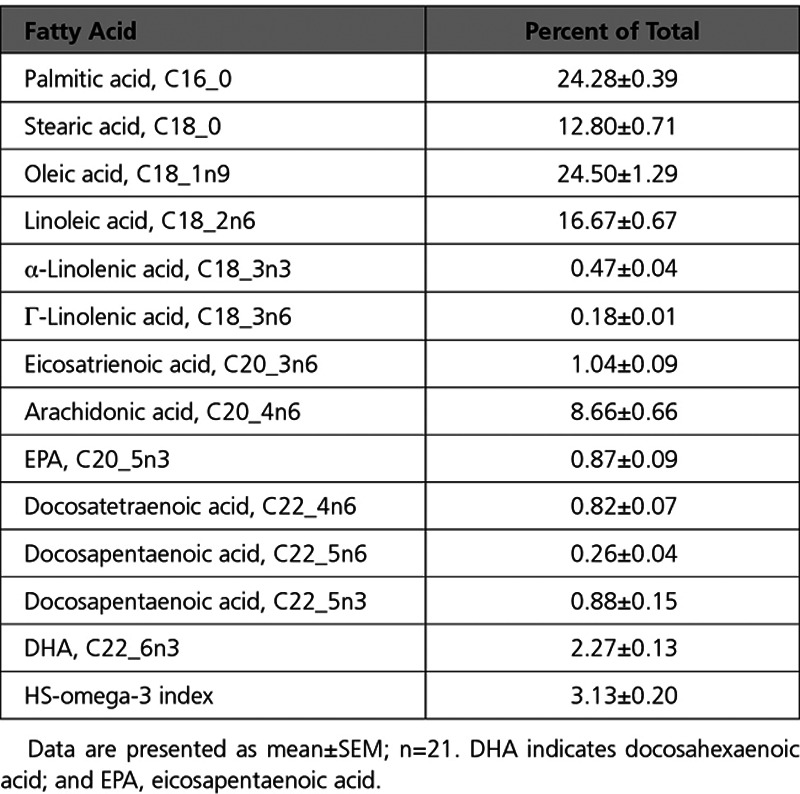
Gas Chromatography Analyses of Noncalcified Aortic Valve Tissue

### Resolvins and Leukotrienes Are Dysregulated in Calcified Aortic Valve Tissue

**Figure 2.**
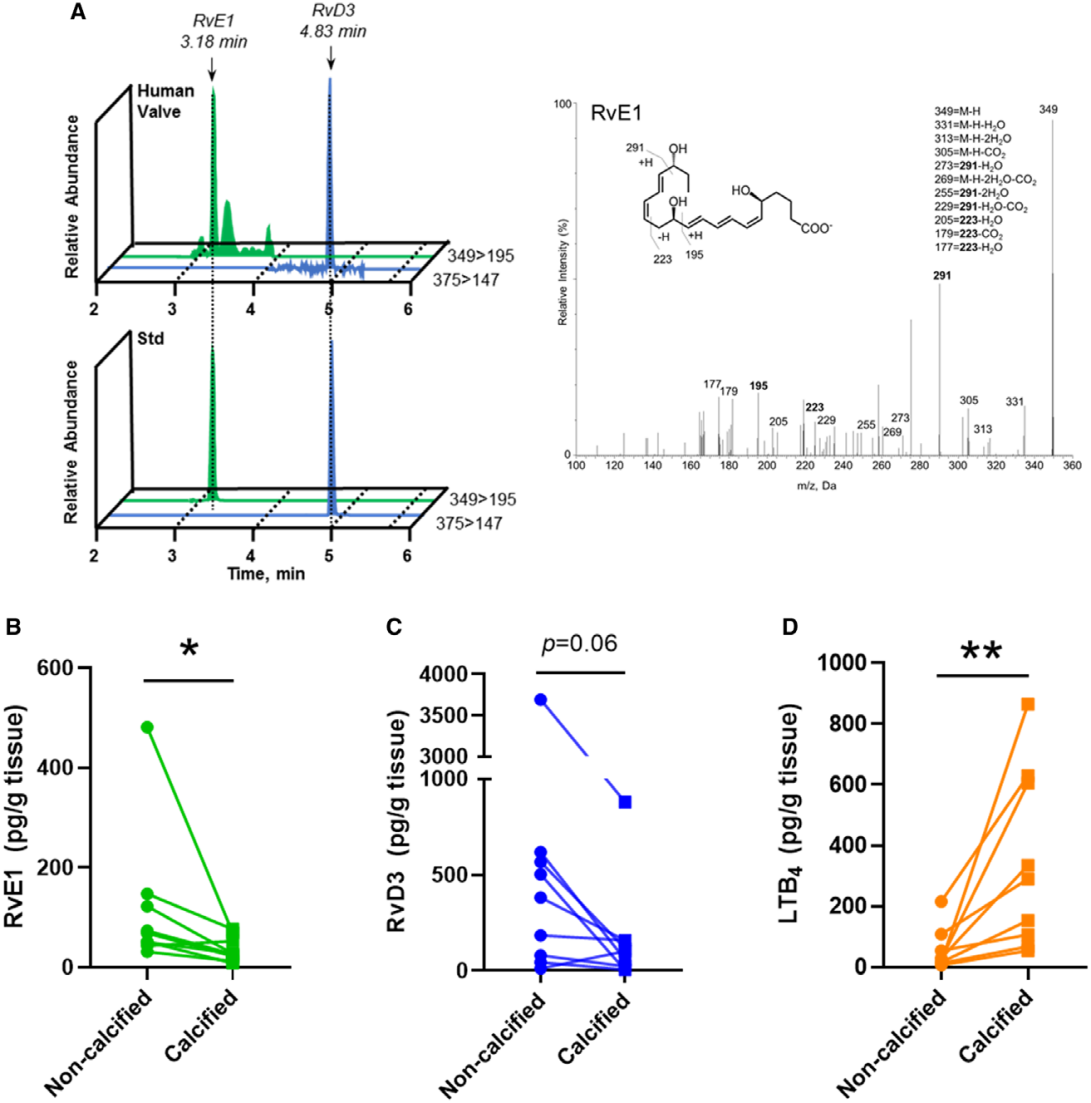
**Resolvin E1 (RvE1) is dysregulated in human calcified valve tissue.** **A**, Representative multiple reaction monitoring chromatograms depicting the relative abundance of RvE1 and resolvin D3 (RvD3; Q1>Q3), and accompanying tandem mass spectrometry spectra used in the identification of RvE1 (inset, diagnostic ions). **B**through **D**, RvE1, RvD3, and leukotriene B4 (LTB_4_) levels from aortic valve tissue determined by liquid chromatography tandem mass spectrometry–based lipid mediator lipidomics. Data are presented as individual values, and statistical significance was evaluated by paired Student *t* test (n=9 vs n=9). M indicates molecular mass. *P<0.05; **P<0.01.

Because the observed changes in aortic valve fatty acid content may alter the substrate availability for downstream lipid mediator formation, a targeted lipid mediator lipidomics by liquid chromatography–tandem mass spectrometry was performed. Here, we identified 2 n-3 PUFA–derived SPMs in human valves, DHA-derived RvD3 (4S,11R,17S-trihydroxy-5Z,7E,9E,13Z,15E,19Z-DHA) and EPA-derived RvE1 (5S,12R,18R-trihydroxy-6Z,8E,10E,14Z,16E-EPA), in both noncalcified and calcified valve tissue. Identification of lipid mediators was performed in accordance with published criteria^14^ that included matching retention time and at least 6 diagnostic ions for each. The tandem mass spectrometry spectra for RvE1 and RvD3 matched those originally identified^17,18^ and are reported as follows for RvD3: 375=M-H; 357=M-H-H_2_O; 339=M-H-2H_2_O; 313=M-H-H_2_O-CO_2_; 295=M-H-2H_2_O-CO_2_; 259=277-H_2_O; 177=195-H_2_O; 159=195-2H_2_O; 147=165-H_2_O) and RvE1 (349=M-H; 331=M-H-H_2_O; 313=M-H-2H_2_O; 305=M-H-CO_2_; 273=291-H_2_O; 269=M-H-2H_2_O-CO_2_; 255=291-2H_2_O; 229=291-H_2_O-CO_2_; 205=223-H_2_O; 179=223-CO_2_; and 177=223-H_2_O. Quantification with multiple reaction monitoring demonstrated that RvE1 levels were significantly lower in calcified compared with noncalcified tissue (Figure 2A and 2B). A similar pattern was observed for RvD3, although the difference between calcified and noncalcified tissue did not reach statistical significance (Figure 2C). In contrast, the proinflammatory n-6 PUFA–derived lipid mediator leukotriene B4 was significantly higher in calcified compared with noncalcified tissue (Figure 2D).

### The RvE1 Receptor ChemR23 Is Expressed in Human Aortic Valves and RvE1 Reduces Calcification in Human Valvular Interstitial Cells In Vitro

**Figure 3.**
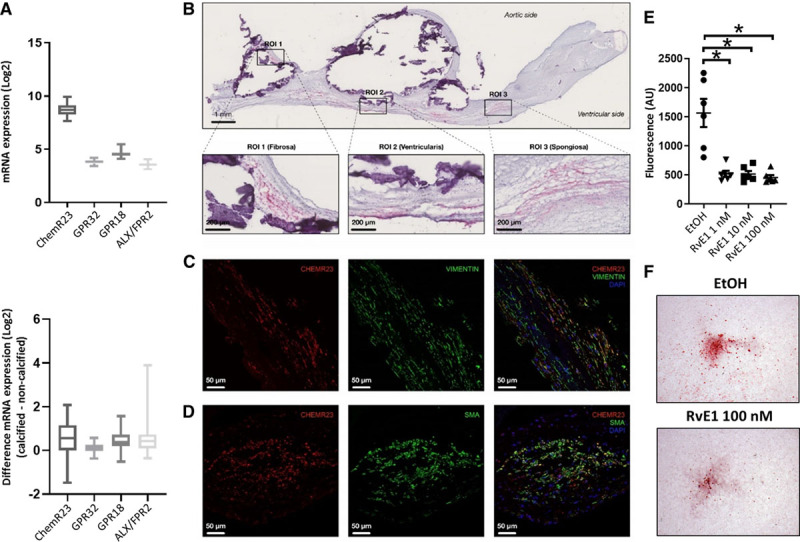
**The resolvin E1 (RvE1) receptor ChemR23 is expressed in human aortic valves. and RvE1 reduces calcification in human valvular interstitial cells (VICs) in vitro.** **A**, Relative abundance of RNA encoding the specialized proresolving lipid mediator receptors ChemR23, GPR18, GPR32, and ALX/FPR2 in noncalcified human valve tissue (n=64; **top**), and difference in gene expression between calcified and noncalcified regions (**bottom**). **B**, Representative photomicrographs of ChemR23 immunohistochemical detection in human aortic valves. White space and dense purple deposits are found at sites of calcified nodules. Higher magnification images of 3 regions of interest (ROIs) display the presence of ChemR23 in the fibrosa (ROI 1), spongiosa (ROI 2), and ventricularis (ROI 3) layers. **C**and**D**, Representative immunofluorescence stainings of human aortic valves showing colocalization of VIC markers (smooth muscle actin [SMA]– and Vimentin-positive cells) and ChemR23. **E**, In vitro effects of RvE1 and quantification by Osteoimage Mineralization Assay of phosphate-induced calcification in VICs after 9 days. Data are presented as individual values, and horizontal lines represent mean±SEM. Statistical significance was evaluated with a mixed-effects ANOVA followed by Holm-Sidak multiple-comparison test; n=3 in duplicates. **F**, Representative photomicrographs of phosphate-treated VICs stained by Alizarin Red. *P<0.05.

To determine whether n-3 PUFA–derived SPMs can exert local effects within the aortic valve, mRNA levels for the SPM receptors ChemR23, GPR18, GPR32, and ALX/FPR2 were explored in noncalcified tissue from 64 human aortic valves. Gene expression analysis revealed ChemR23, encoding the RvE1 receptor, as the predominant SPM receptor in human aortic valves (Figure 3A, top), whereas the differential expression of the same receptors between calcified and noncalcified regions was similar (Figure 3A, bottom). Moreover, ChemR23 protein expression detected by immunohistochemistry was widely distributed in the fibrosa, spongiosa, and ventricularis layers of human aortic valves and in calcified and noncalcified regions (Figure 3B), colocalizing with the valvular interstitial cell (VIC) markers smooth muscle actin and vimentin by immunofluorescence (Figure 3C and 3D).

Given the expression of ChemR23 in VICs and the decreased levels of RvE1 in calcified valve tissue, we next studied the effect of RvE1 on VIC calcification induced by a high-phosphate media. RvE1 significantly decreased calcification of human VICs compared with vehicle (Figure 3E and 3F).

### n-3 PUFA Concentrations Are Higher and n-6 PUFA Concentrations Are Lower in Aortic Valve Leaflets of Fat-1^tg^×Apoe^−/−^ Mice Compared With Apoe^−/−^ Mice

**Figure 4.**
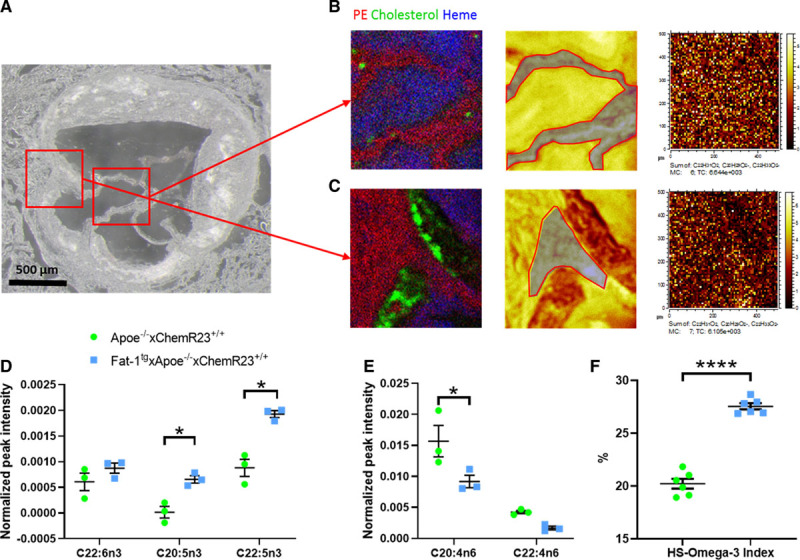
**Omega-3 polyunsaturated fatty acids (PUFAs) are higher and n-6 PUFAs are lower in valve leaflets of Fat-1^tg^×Apoe^−/−^ compared with Apoe^−/−^ mice.** **A**, Optical micrograph of an aortic root section of a Fat-1^tg^×Apoe^−/−^ mouse. Red squares indicate areas (500×500 µm^2^) for focused analyses of valve leaflets and valve insertion, respectively. **B**and **C**, Time-of-flight secondary ion mass spectrometry (TOF-SIMS) data from 2 different regions of the valve leaflet area with ion images of (**left**) overlay image of phosphatidylethanolamine (PE) in red, cholesterol in green, and heme in blue (**left**); total ion image with region of interest (ROI) used for extraction of mass spectrum of valve leaflet indicated in gray (**middle**); and image of the added signal intensity of eicosapentaenoic acid (EPA; C20:5n3), docosahexaenoic acid (DHA; C22:6n3), and docosapentaenoic acid (DPA, C22:5n3; **right**). Brighter pixels in the ion images correspond to higher signal intensities. **D**, n-3 PUFAs (DHA, EPA, and DPA) and (**E**) n-6 PUFAs (arachidonic acid [C20:4n6] and adrenic acid [C22:4n6]) normalized TOF-SIMS signal intensities (n=3 animals per group; each observation is the average of 3 independent leaflet regions at different valve levels) in mass spectra acquired from valve leaflets. **F**, HS-omega-3 index in ventricular myocardium measured by gas chromatography in Apoe^−/−^ compared with Fat-1^tg^×Apoe^−/−^ mice (n=6 per group). Data are presented as individual values with horizontal lines representing mean±SEM. Statistical significances were evaluated with either a Student *t* test or a 2-way repeated measures ANOVA followed by Holm-Sidak multiple-comparison test. *P<0.05. ****P<0.0001.

To test the applicability of the beneficial effects of n-3 PUFA on aortic valve disease (AVD) under different conditions, we established the expression of the *C elegans* Fat-1^tg^ in Apoe^−/−^ mice, which enables the endogenous production of n-3 PUFAs. n-3 PUFAs and n-6 PUFAs were measured in aortic valve leaflet regions of Fat-1^tg^×Apoe^−/−^ and Apoe^−/−^ mice using time-of-flight secondary ion mass spectrometry. Spatially resolved mass spectrometric data were obtained by time-of-flight secondary ion mass spectrometry analysis of tissue sections containing the aortic valve region. The data were used to generate ion images, which display the lateral distribution of specific molecular species within the analysis area and mass spectra from selected structures on the tissue section surface, including different valve leaflet regions. The aortic root, identified by optical microscopy (Figure 4A), exhibited presence of phosphatidylethanolamine on the tissue surface, cholesterol in atherosclerotic plaque regions, and heme in the tissue-free area, indicating blood remnants (Figure 4B and 4C, left). Spatially resolved fatty acid data were then extracted by generating mass spectra from regions of interest exclusively corresponding to valve leaflet regions (Figure 4B and 4C, center and right).

Analysis of the fatty acid signal intensities in all mass spectra of the central regions of the valve leaflets demonstrated significantly higher relative concentrations of n-3 PUFAs (EPA, docosapentaenoic acid) and significantly lower relative concentrations of n-6 PUFAs (arachidonic acid) in Fat-1^tg^×Apoe^−/−^ mice compared with Apoe^−/−^ mice (Figure 4D and 4E, respectively). In addition, and consistent with the previous observation, Fat-1^tg^×Apoe^−/−^ mice exhibited a significant increase in HS-omega-3 index in ventricular myocardium (Figure 4F). Full gas chromatography analysis in ventricular myocardium of Apoe^−/−^ and Fat-1^tg^×Apoe^−/−^ is shown in Table IV in the Data Supplement.

### Fat-1^tg^ Halts Whereas Targeted Deletion of ChemR23 Increases Echocardiographic Progression in Apoe^−/−^ Mice

**Figure 5.**
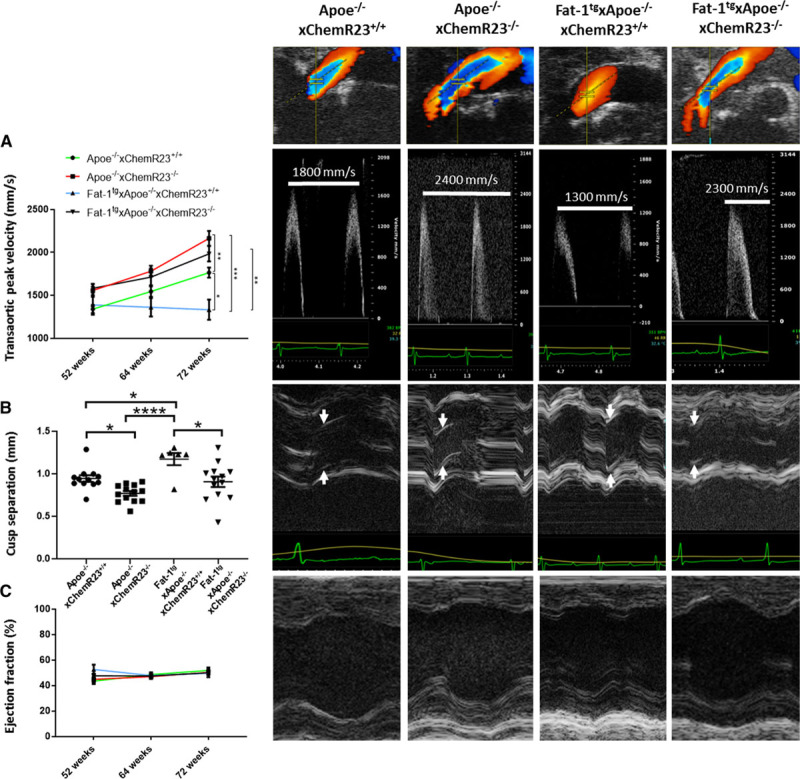
**Fat-1^tg^ halts whereas targeted deletion of ChemR23 increases echocardiographic progression in Apoe^−/−^ mice.** **A**, Transaortic peak velocity, (**B**) cusp separation, and (**C**) ejection fraction in 52-, 64-, and 72-week-old Apoe^−/−^×ChemR23^+/+^ (n=12), Apoe^−/−^×ChemR23^−/−^ (n=13), Fat-1^tg^×Apoe^−/−^×ChemR23^+/+^ (n=6), and Fat-1^tg^×Apoe^−/−^×ChemR23^−/−^ (n=13) mice. Representative color Doppler, Doppler, and M-mode tracings are shown in 72-week-old mice. Data are presented either as individual values with horizontal lines representing mean±SEM or as mean±SEM. Statistical significances were evaluated with either a 1- or 2- way repeated measures ANOVA followed by Holm-Sidak multiple-comparison test. Results are pooled from 2 independent experiments. *P<0.05; **P<0.01; ***P<0.001; ****P<0.0001.

Transaortic peak velocity (Vmax) and aortic valve cusp separation were evaluated at 52, 64, and 72 weeks of age. Apoe^−/−^ mice had progressively increased Vmax with age (Figure 5A) to velocities above normal mice (>1.5 m/s) at 72 weeks.^19^ Fat-1^tg^×Apoe^−/−^ mice exhibited a significantly reduced Vmax (Figure 5A), a significantly increased aortic valve cusp separation (Figure 5B), and reduced left ventricular mass (Table V in the Data Supplement) at 72 weeks compared with nontransgenic Apoe^−/−^ mice (Figure 5B). The full echocardiographic analysis determined at the study end point at 72 weeks is shown in Table V in the Data Supplement. There were no significant differences in heart rate between the different groups at any age (Tables V and VI in the Data Supplement).

On the basis of the results obtained in the analysis of human aortic valves (see above and Figures 1 through 3), the RvE1/ChemR23 axis was abrogated by genetic deletion of ChemR23 in the Apoe^−/−^ model to determine the mechanisms through which n-3 PUFAs induced the beneficial effects on the aortic valve. Apoe^−/−^×ChemR23^−/−^ mice exhibited significantly increased Vmax and reduced cusp separation compared with Apoe^−/−^×ChemR23^+/+^ mice, pointing to the ChemR23 receptor as a transducer of aortic valve protection (Figure 5A and 5B).

To establish whether the beneficial effects of an increased n-3 PUFA pathway were transduced through ChemR23, Fat-1^tg^ was introduced also in the Apoe^−/−^×ChemR23^−/−^ mice. The beneficial effects observed in Fat-1^tg^×Apoe^−/−^ (reduced Vmax and increased aortic cusp separation) were completely abolished in Fat-1^tg^×Apoe^−/−^×ChemR23^−/−^ mice (Figure 5A and 5B), suggesting a direct effect of n-3 PUFA–derived RvE1 signaling through ChemR23 in the progression of the disease. Vmax inversely correlated with cusp separation (adjusted *R*^2^= 0.61, *P*<0.0001; partial *R*^2^= 0.36, *P*<0.0001), supporting that the increased Vmax was a result of reduced cusp separation. The ejection fractions were in line with previous reports,^20^ and no significant differences were observed between the 4 groups (Figure 5C). No regurgitations were observed in any mouse. Finally, echocardiographic evaluation of control (Apoe^+/+^) ChemR23^+/+^, ChemR23^−/−^, Fat-1^tg^×ChemR23^+/+^, and Fat-1^tg^×ChemR23^−/−^ mice did not reveal either increased gradients or reduced cusp separation, and no any apparent differences were observed between groups and time (Figure IA through ID in the Data Supplement).

### Fat-1^tg^ Reduces Aortic Valve Leaflet Area and Targeted Deletion of ChemR23 Increases Aortic Valve Leaflet Area in Apoe^−/−^ Mice

**Figure 6.**
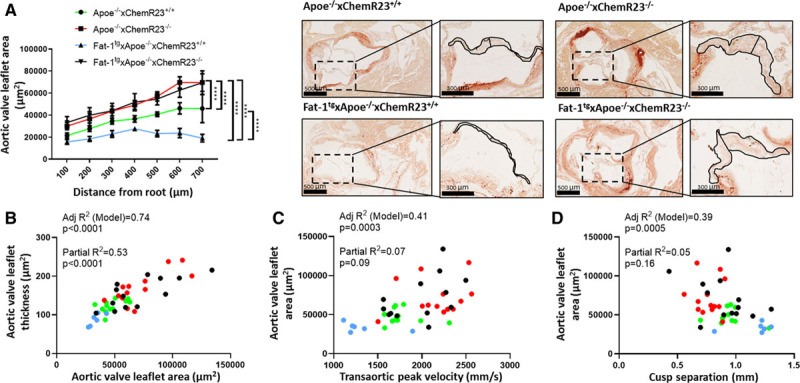
**Fat-1^tg^ reduces aortic valve leaflet area and targeted deletion of ChemR23 increases aortic valve leaflet area in Apoe^−/−^ mice.** **A**, Aortic valve leaflet area in 72-week-old Apoe^−/−^×ChemR23^+/+^ (n=12), Apoe^−/−^×ChemR23^−/−^ (n=13), Fat-1^tg^×Apoe^−/−^×ChemR23^+/+^ (n=10), and Fat-1^tg^×Apoe^−/−^×ChemR23^−/−^ (n=13) mice and representative photomicrographs. Data are presented as mean±SEM. Statistical significance was determined with a 2-way ANOVA followed by Holm-Sidak multiple-comparison test. ****P<0.0001. **B**, Adjusted *R*^2^ and partial *R*^2^ for the correlation between aortic valve leaflet area and aortic valve leaflet thickness. **C**, Adjusted *R*^2^ and partial *R*^2^ for the correlation between transaortic peak velocity and aortic valve leaflet area. **D**, Adjusted *R*^2^ and partial *R*^2^ for the correlation between cusp separation and aortic valve leaflet area. Apoe^−/−^×ChemR23^+/+^ (n=12), Apoe^−/−^×ChemR23^−/−^ (n=13), Fat-1^tg^×Apoe^−/−^×ChemR23^+/+^ (n=6), and Fat-1^tg^×Apoe^−/−^×ChemR23^−/−^ (n=13) mice for all correlations.

In addition to the echocardiographic measures of aortic velocities and aortic cusp separation, morphological analysis supported beneficial effects of the Fat-1^tg^ through ChemR23 on aortic valve leaflet area. Fat-1^tg^×Apoe^−/−^ mice exhibited reduced aortic valve leaflet area compared with nontransgenic mice in the presence but not in the absence of ChemR23. In contrast, Apoe^−/−^×ChemR23^−/−^ mice exhibited a significant increase in aortic valve leaflet area compared with Apoe^−/−^×ChemR23^+/+^ mice in both the presence and absence of the Fat-1^tg^ (Figure 6A). Moreover, aortic valve leaflet area was significantly correlated with aortic valve leaflet thickness (Figure 6B), as well as with the echocardiographic measures in terms of Vmax (Figure 6C) and inversely with cusp separation (Figure 6D) in adjusted models. The nonsignificant partial correlations for the quantitative variables supported genotype as the predictor for the observed associations (Figure 6B through 6D).

### Fat-1^tg^ Reduces Leaflet Calcification and Targeted Deletion of ChemR23 Increases Leaflet Calcification in Apoe^−/−^ Mice

**Figure 7.**
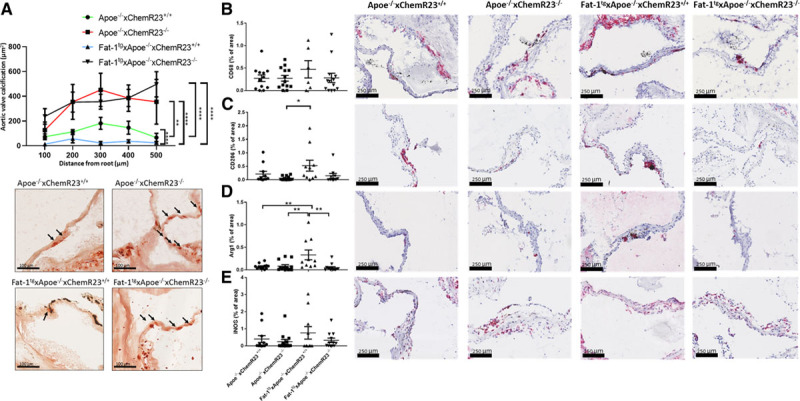
**Fat-1^tg^ reduces leaflet calcification and induces M2 macrophage polarization and targeted deletion of ChemR23 increases leaflet calcification in Apoe^−/−^ and Fat-1^tg^ mice.** **A**, Quantification of Alizarin Red–stained calcification in 72-week-old Apoe^−/−^×ChemR23^+/+^ (n=12), Apoe^−/−^×ChemR23^−/−^ (n=13), Fat-1^tg^×Apoe^−/−^×ChemR23^+/+^ (n=10), and Fat-1^tg^×Apoe^−/−^×ChemR23^−/−^ (n=13) and representative photomicrographs. **B**, Representative photomicrographs and quantification of leaflets stained with antibodies against the macrophage marker CD68, (**C**) CD206, (**D**) arginase 1 (Arg1), and (**E**) inducible nitric oxide synthase (iNOS) normalized to total aortic valve leaflet area in 72-week-old Apoe^−/−^×ChemR23^+/+^ (n= 11-12), Apoe^−/−^×ChemR23^−/−^ (n=13), Fat-1^tg^×Apoe^−/−^×ChemR23^+/+^ (n= 6-10), and Fat-1^tg^×Apoe^−/−^×ChemR23^−/−^ (n= 10-13). Statistical significance was determined with a 1- or 2-way repeated measures ANOVA followed by Holm-Sidak multiple-comparison test. *P<0.05; **P<0.01; ****P<0.0001.

Because calcification is a main component in human AVS and because n-3 PUFA content and RvE1 formation are decreased in calcified compared with noncalcified regions of human aortic valves (see above and Figures 1 through 3), aortic valve leaflet calcification of the 4 groups of mice was subsequently analyzed by Alizarin Red staining. These analyses revealed that Fat-1^tg^×Apoe^−/−^×ChemR23^+/+^ mice exhibited a significantly reduced calcification compared with Apoe^−/−^×ChemR23^+/+^ mice. In addition, Apoe^−/−^×ChemR23^−/−^ mice exhibited significantly higher leaflet calcification compared with Apoe^−/−^×ChemR23^+/+^ in both the absence and presence of Fat-1^tg^ (Figure 7A). Moreover, leaflet calcification correlation with aortic valve leaflet area (adjusted *R*^2^= 0.38, *P*=0.0009; partial *R*^2^= 0.02, *P*=0.35), Vmax (adjusted *R*^2^= 0.49, *P*<0.0001; partial *R*^2^= 0.001, *P*=0.83), and cusp separation (adjusted *R*^2^= 0.37, *P*=0.0011; partial *R*^2^= 0.001, *P*=0.82) was significant in adjusted models, whereas the nonsignificant partial *R*^2^ supported genotype as the predictor for the observed associations. No significant differences in blood cell counts were observed between the different groups (Table V in the Data Supplement).

### Fat-1^tg^ Induces M2 Macrophage Polarization

Further characterization was performed to establish how the observed hemodynamic and morphological effects were related to changes in the inflammatory response. Because macrophages represent a major immune cell population in the stenotic valves and because macrophage polarization is a crucial step in the resolution of inflammation, immunohistochemistry for different macrophage markers in the mouse aortic valves was performed. Whereas the proportion of leaflet area containing CD68 did not significantly differ between the groups (Figure 7B), a significantly increased proportion of the M2 macrophage markers CD206 and arginase 1 was observed in Fat-1^tg^×Apoe^−/−^ mice (Figure 7C and 7D), which was lost in mice lacking ChemR23 (Figure 7D). In contrast, the M1 macrophage marker inducible nitric oxide synthase was not significantly different between the 4 groups (Figure 7E).

### ChemR23 Correlates With M2 Macrophage Markers in Human Aortic Valves

In line with the findings in murine valves, we found a strong correlation between ChemR23 and CD206 mRNA levels in both calcified and noncalcified parts of human aortic valves (Table VIII in the Data Supplement). In addition, ChemR23 was positively and significantly correlated with the M2 macrophage markers CD163, CD209, CD200R1, and heme oxygenase 1 (Table VIII in the Data Supplement).

### Targeted Deletion of ChemR23 Induces Higher Vmax and Increased Thickening of the Aortic Valve After Aortic Valve Wire Injury

Because n-3 PUFAs reduced aortic valve obstruction, thickness, and calcification by means of ChemR23 in the context of hypercholesterolemia, we finally evaluated the effect of ChemR23 deletion of aortic valve hemodynamics and morphology in the absence of hypercholesterolemia. A second model of AVD was therefore established for this study by using normolipidemic ChemR23^+/+^ (C57BL/6J) and ChemR23^−/−^ mice and applying the protocol of aortic valve wire injury introduced by Honda et al^21^ and called mild injury by Niepmann et al.^22^ Consistent with the aforementioned results in Apoe^−/−^ mice, the Vmax was significantly higher in ChemR23^−/−^ mice 16 weeks after aortic valve injury compared with ChemR23^+/+^ mice, with no significant difference in ejection fraction between the 2 groups. Tissue morphology analysis revealed that ChemR23^−/−^ mice exhibited significantly increased aortic valve leaflet thickness compared with ChemR23^+/+^ mice (Figure IIA through IIC in the Data Supplement). No significant differences in blood cell counts were observed between the 2 genotypes (Table IX in the Data Supplement).

## Discussion

This is the first study to indicate beneficial effects of n-3 PUFAs inhibiting aortic valve thickening and calcification and retarding AVD progression. This conclusion was based on several observations as discussed below. First, starting from observational analysis of human aortic valves, we demonstrated that decreased aortic valve n-3 PUFA levels were associated with valve calcification, a specific transcriptomic profile, M2 macrophage markers, and a trend toward faster progression into severe AVS. Second, we detected high levels of the n-3 PUFA–derived SPM RvE1 and its receptor ChemR23 in noncalcified valve tissue and showed anticalcifying effects of RvE1 on VICs. Third, by means of a combined strategy using different genetic targeting and several murine AVD models, we identified for the first time the presence of n-3 PUFAs in the mouse aortic valve and that the mechanisms behind the beneficial hemodynamic effects were mediated by reduced leaflet area, calcification, and increased M2 macrophage polarization, transduced through the RvE1 receptor ChemR23. From these results, the n-3 PUFA/RvE1/ChemR23 axis emerges as a novel therapeutic opportunity for AVD.

Although the recently reported beneficial effects of high-dose EPA ethyl ester on cardiovascular events^5,23^ have renewed interest in n-3 PUFAs in cardiovascular prevention, their effects on AVS have remained unknown. By a combined lipidomic and transcriptomic approach, we demonstrate here the n-3 PUFA incorporation into human aortic valves, in the same range as previously reported in red blood cells^24^ and myocardial biopsies,^25^ being associated with a specific gene expression pattern. In addition, valvular n-3 PUFA levels tended to be lower in a group of patients with a history of more rapid progression from moderate to severe AVS, providing a first indication of a role for valvular n-3 PUFA levels in the development of AVD. A link to calcification has previously been suggested on the basis of the inverse association between serum n-3 PUFA levels and coronary artery calcification,^26^ and the present study extends those observations to valvular heart disease by showing lower n-3 PUFA incorporation in calcified compared with noncalcified human aortic valve tissue.

Using Apoe^−/−^ mice as a model of AVD with the transgenic expression of the *C elegans* Fat-1 gene,^15^ which enables the endogenous synthesis of n-3 PUFAs, we show increased incorporation of n-3 PUFAs and a decreased incorporation of n-6 PUFAs in the aortic valve and in the myocardium. Furthermore, we demonstrate protective effects of n-3 PUFAs in murine AVD. Echocardiographic assessment showed that n-3 PUFAs reduced Vmax and increased cusp separation and that this was associated with changes in valve morphology in terms of a reduction in aortic valve leaflet thickness and area. Moreover, the progressive valvular calcification characteristic of Apoe deficiency^27,28^ was reduced in Fat-1 transgenic Apoe^−/−^ mice. Similar results have been found in klotho mutant mice in which EPA significantly reduced arterial calcification, decreased oxidative stress, and downregulated NADPH oxidase-4 expression and activity.^29^

Under certain conditions, n-3 PUFAs are converted into resolvins, a group of bioactive lipid metabolites with proresolving actions.^6^ The present study is the first to identify resolvins in human aortic valves, of which RvE1 levels significantly decreased in calcified regions. Other lipid mediator pathways with opposed actions such as proinflammatory leukotrienes are increased as aortic valve calcification progresses,^30,31^ which was confirmed for leukotriene B4 in the present study, which also reinforces that calcified valve tissue remains biologically active. As a consequence, the resolvin/leukotriene ratio, a marker of nonresolving inflammation,^32,33^ was lower in calcified regions of human aortic valves, pointing to a local nonresolved inflammation in valve calcification. RvE1 signals through ChemR23^34^ and has been shown, for example, to reduce atherosclerosis^9^ and intimal hyperplasia.^10,35^ RvE1-induced effects on macrophages include decreasing inflammation, inhibiting oxidized low-density lipoprotein uptake, and increasing phagocytosis.^9^ Those proresolving actions are stimulated by a macrophage polarization toward an M2-like or intermediate phenotype.^7^ Consistent with this, ChemR23 in human aortic valves strongly correlated with M2 markers. In addition, we show that Fat-1^tg^ mice exhibited an increased M2 macrophage polarization in murine valves and that those effects were lost in mice lacking ChemR23. In addition to macrophages, RvE1 signaling through ChemR23 on vascular smooth muscle cells diminishes vascular calcification by lowering the expression of bone morphogenetic protein 2 in vascular smooth muscle cells.^11^ An extrapolation of the latter results to valve calcification can be anticipated on the basis of the dominant valvular ChemR23 expression compared with other SPM receptors, the ChemR23 colocalization with VICs, and the reduced VIC calcification by RvE1 demonstrated in the present study. Taken together, these results suggest that valvular n-3 PUFAs can serve as a substrate for resolvin formation and point to RvE1 signaling through ChemR23-transducing protective effects through both inflammatory and structural valve cells.

In support of therapeutic potential for the latter findings, ChemR23 deletion in Apoe^−/−^ mice accelerated AVD, exhibiting increased Vmax, reduced cusp separation, and increased aortic valve leaflet area and calcification in the present study. We and others have previously established increased RvE1 formation in mice after exogenous n-3 PUFA administration^9^ and Fat-1^tg^ insertion.^36,37^ To determine whether the observed beneficial effects of n-3 PUFAs were mediated by RvE1 signaling through ChemR23, the Fat-1^tg^ was bred into Apoe^−/−^ and ChemR23^−/−^ knockout mice. These experiments confirmed that the observed beneficial effects of n-3 PUFA were completely abolished in mice lacking ChemR23. Moreover, the exacerbated calcification in Apoe^−/−^×ChemR23^−/−^ mice was not reversed by Fat-1^tg^ in the Fat-1^tg^×Apoe^−/−^×ChemR23^−/−^ mouse construct. Taken together, these results demonstrate that the beneficial effects of n-3 PUFA on AVD were driven through ChemR23. However, it cannot be excluded that other n-3 PUFA–derived SPMs also are involved in human AVS. For example, we detect RvD3 formation in human valves, a lipid mediator that has potent anti-inflammatory and proresolving actions that include limiting cytokine production and neutrophil infiltration and enhancing macrophage phagocytosis,^38^ which may play an important role in the development of AVS.

To validate that the beneficial effects of ChemR23 signaling were independent of hyperlipidemia and atherosclerosis, we finally used a normolipidemic model by direct aortic valve wire injury in ChemR23^+/+^ and ChemR23^−/−^ mice. Indeed, these experiments demonstrated an increased Vmax and aortic valve leaflet thickness in absence of ChemR23, hence confirming that the beneficial effects on AVD mediated through ChemR23 signaling were reproducible also under normolipidemic condition and in the absence of atherosclerosis.

The strengths of the present study include the exploration of the n-3 PUFA/RvE1/ChemR23 axis in human tissue and cells and in several independent animal models. It should be acknowledged, however, that observational studies in human valves cannot establish causality for the association of valvular n-3 PUFA content with valve calcification and aortic stenosis progression. Likewise, although our murine models present valve thickening, calcification, reduced cusp separation, and hemodynamic signs of accelerated aortic valve flow, it is as yet not known how improvement of those parameters translates into a clinical benefit for patients with AVS. In fact, previous preclinical studies on AVD in animal models have shown other potential treatments for the disease, for example, by lowering plasma cholesterol with the use of statins^39,40^ or by genetic inactivation of the *mttp* gene.^41^ However, those results did not translate into reduced aortic stenosis progression in clinical trials.^42–44^ It should also be noted that the models used here did not attain Vmax equivalent to human severe AVS. Technical limitations should also be considered for the use of pulse-wave Doppler with angle correction, which may not optimally capture maximal transvalvular velocities. Although further assessment in more severe AVD models would be of additional value to establish the role of the n-3 PUFA/RvE1/ChemR23 axis over the full disease continuum, the degree of AVD in the models used in the present study may be equal to the mild to moderate AVD that would represent a therapeutic window for a potential medical treatment. Furthermore, the replication of the observations in 2 independent murine AVD models in the present study under different conditions in which wild-type control mice did not exhibit signs of AVD reinforces the reproducibility of the observations.

### Conclusions

This study demonstrates for the first time that increased n-3 PUFA content in human aortic valves is associated with less calcification and a specific transcriptomic pattern and that aortic valve n-3 PUFAs decrease AVD in vivo, being consistent for both hemodynamic and morphological criteria and across different murine models. We further decipher the mechanism being mediated through the RvE1 receptor ChemR23. From the translational discoveries presented in this study, the n-3 PUFA–derived RvE1 and its receptor ChemR23 emerge as a key axis in the inhibition of AVD progression. Taken together with the recent clinical trial demonstrating reduced cardiovascular risk with high-dose EPA,^5^ the results of the present study urge that the n-3 PUFA/RvE1/ChemR23 axis be clinically evaluated as a novel potential therapeutic opportunity to slow AVD progression and to improve the prognosis for these patients.

## Acknowledgments

The authors thank Omegametrix GmbH (Planegg, Germany) for performing the fatty acid analyses and Carlos Labat and Abdul Rashid Qureshi for the statistical analysis support.

## Sources of Funding

This study was supported by the Swedish Research Council (grant 2019-01486), the Swedish Heart and Lung Foundation (grant 20180571), King Gustaf V and Queen Victoria Freemason Foundation, the Stockholm County Council (grant 20170365), and Marianne and Marcus Wallenberg Foundation (grant 2015.0104). G. Artiach was supported by the Swedish Heart and Lung Foundation (grant 20180572). Dr Thul was supported by the Swedish Heart and Lung Foundation (grant 20184251) and through a research fellowship from the Deutsche Forschungsgemeinschaft (grant MU 3857/1–1). Dr Laguna-Fernandez was supported by a fellowship from the Center of Excellence for Research on Inflammation and Cardiovascular Disease (CERIC Linnaeus Program, grant 349-2007-8703) and funds from Nanna Svartz Fond, Fredrik och Ingrid Thurings Stiftelse, Stiftelsen för Gamla Tjänarinnor, and the Foundation for Geriatric Diseases at Karolinska Institutet. Dr Arnardottir was supported by the Swedish Heart and Lung Foundation (grant 20170311), the Foundation for Geriatric Diseases at Karolinska Institutet, and a European Union Horizon 2020 Marie Skłodowska-Curie fellowship (under grant agreement 656817).

## Disclosures

None.

## Supplemental Materials

Expanded Methods

Data Supplement Figures I–II

Data Supplement Tables I–IX

References 45–53

## Supplementary Material


