# Supplementary material for: Omega-3 Polyunsaturated Fatty Acids Decrease Aortic Valve Disease Through the Resolvin E1 and ChemR23 Axis
Source: Circulation. 2020 Jun 8;142(8):776–89. doi: 10.1161/CIRCULATIONAHA.119.041868 (PMC7439935; doi:10.1161/CIRCULATIONAHA.119.041868)
Supplement: Supplementary file 1 [file cir-142-776-s001.pdf]

Supplemental material

Supplemental materials and methods

Fatty Acid Content

For fatty acid composition analyses, samples of non-calcified or calcified regions of human aortic valves, and ventricular myocardium from mouse were used. Human and murine samples were homogenized in liquid nitrogen using a biopulverizer (Biospec). Pulverized tissues were transferred to stabilizing filter papers and analyzed at Omegametrix (Munich, Germany) as previously described[45]. In brief, gas chromatography was performed in a GC2010 gas chromatograph (Shimadzu) equipped with a 100-m SP2560 column (Supelco) and using hydrogen as carrier gas. Fatty acids were identified by comparison with standards. Results are given as a percentage of total identified fatty acids after response factor correction. The HS-Omega-3 Index is defined as the combined percentage of eicosapentaenoic acid (EPA) and docosapentaenoic acid (DHA) of total fatty acids in tissue, and it represents an individual's status in these two marine n-3 PUFA[46].

Human AVS Progression Analysis

Hemodynamic aortic valve stenosis (AVS) progression of patients undergoing aortic valve replacement was assessed according to the 2017 ESC/EACTS Guidelines for the management of VHD[47]. In brief, patients with ≥ 0.3 m/s/year increase in transaortic peak velocity (Vmax) were considered AVS fast progressors, and patients with < 0.3 m/s/year increase in Vmax were considered AVS slow progressors. 11 Patients with ≥2 echocardiographic measurements before surgery were included, with > 6 months in between echocardiographies.

RNA Extraction and Quality Assessment

Total RNA from non-calcified tissue of human aortic valves was isolated with the RNeasy Lipid Tissue Mini kit (Qiagen, Hilden, Germany). RNA concentration and quality were assessed using a NanoDrop (Thermo Scientific, Waltham, MA, USA) and a 2100 Bioanalyzer (Agilent, Santa Clara, CA, USA), respectively. Valve gene expression data was obtained using Gene Chip Affymetrix human transcriptome 2.0 (HTA 2.0 arrays, Santa Clara, CA, USA) and normalized with Signal space transformation-robust multi-chip analysis (SST-RMA) and log2-transformed using Expression Console (Affymetrix, Santa Clara, CA, USA) as previously described[48]. All recommended Quality Control assessments were carried, without resulting exclusion of samples. Affymetrix probe names used in the study: TC12001933.hg.1 (ChemR23), TC19000766.hg.1 (GPR32), TC13000828.hg.1 (GPR18), TC19000788.hg.1 (ALX/FPR2), TC10000132.hg.1 (CD206), TC06000983.hg.1 (Arg1), TC12001162.hg.1 (CD163), TC19001118.hg.1 (CD209), TC22000259.hg.1 (HMOX1), TC03001654.hg.1 (CD200R1).

Transcriptomics analysis

Clinical data and the normalized log2 expression values were imported into Qlucore Omics Explorer (QOE 3.4) bioinformatics software (http://www.qlucore.com). Samples were stratified based on the HS-Omega-3 index defined as low if < median and high if value ≥ median. A variance filter and a t-test with cut-off values 0.02 and 0.001 respectively were applied, age and sex adjusted. The results were visualized with a principal component analysis. Furthermore, to assess pathways enriched in samples with high or low HS-Omega-3 index, Gene Set Enrichment Analysis (GSEA) was performed according to the Broad Institute GSEA user guide (http://software.broadinstitute.org/gsea/) using QOE 3.4 with a two-group

comparison, age and sex adjusted. Equal GSEA-settings and cut-off values were used in both calcified and non-calcified tissue. Q-values were adjusted for multiple testing using the FDR method of Benjamini-Hochberg. Spearman correlations were used for transcriptomic analysis of non-calcified and calcified valve tissue.

Time-of-flight secondary mass spectrometry (TOF-SIMS)

Time-of-flight secondary ion mass spectrometry (TOF-SIMS) was used to investigate presence of n-3 PUFA and n-6 PUFA in Fat-1^{tg}xApoe$^{-/-}$ and Apoe$^{-/-}$ mice in the aortic valves. TOF-SIMS is a mass spectrometric method that provides molecular information of solid sample surfaces at spatial resolutions down to the submicrometer regime[49]. During analysis, a focused beam of high-energy (primary) ions is directed onto the sample surface and secondary ions, which are emitted from the sample as a result of the primary ion collisions with the surface, are detected in a TOF mass analyzer to produce a mass spectrum of the molecular species present on the sample surface. Scanning the primary ion beam across a specified analysis area and recording separate mass spectra from each pixel enables imaging of specific ions and/or extraction of mass spectra from selected regions of interest (ROIs) within the analysis area.

Tissue sections of mouse aortic valves were analyzed under static SIMS conditions in a TOFSIMSIV instrument (IONTOF GmbH, Germany) using 25 keV Bi_3^+ primary ions and low-energy electron flooding for charge compensation. Positive and negative ion data were recorded with the instrument optimized for high mass resolution ($m/\Delta m \approx 5{,}000$) at a spatial resolution of 3-5 µm. The analysis included three tissue sections from each of three Fat-1^{tg}xApoe$^{-/-}$ and three Apoe$^{-/-}$ mice (i.e., nine tissue sections in total from each mouse type). The tissue sections (16 µm thick) were prepared using a cryostat at -20°C and placed on laboratory slides (Superfrost, Thermo Scientific, Waltham, MA, USA). Sections were shipped on dry ice and stored at -20°C until TOF-SIMS analysis (maximum 10 days). Prior to TOF-SIMS

analysis, the tissue sections were freeze dried by placing the glass slides inside the load lock of the TOF-SIMS instrument and evacuating to $10^{-6} – 10^{-5}$ mbar for about 30 min while keeping the temperature at approximately -20°C. Finally, the sample temperature was quickly raised to room temperature by placing the glass slides on a metal block under dry conditions, to avoid condensation on the sample surface.

For each individual tissue section, TOF-SIMS data was acquired over a large analysis area (3x3 mm^2), covering the entire aortic valve region, and several smaller (500x500 μm^2) areas focusing on different tissue types, including different regions of the aortic valve. For evaluation of the fatty acid distributions, negative ion spectra were generated from ROIs selected to explicitly represent valve leaflet regions (using the TOF-SIMS instrument software, SurfaceLab v6.8). The integrated, background-subtracted and dead-time corrected signal intensities of peaks corresponding to the most abundant, and for this study most relevant, fatty acids (C16:1, C16:0, C18:2, C18:1, C18:0, C20:5, C20:4, C20:3, C20:2, C20:1, C20:0, C22:4, C22:5, C22:6) were extracted from these spectra. The PUFA signal intensities in each spectrum were then normalized to the added signal intensity of all included fatty acids. The fatty acid data in Fig 4 are given as mean ± standard error of the mean (SEM) in n=3 mice where each observation results as the average of 3 independent different sections. The overlay ion images in Fig 4 show the signal intensity of peaks identified as $C_5H_{11}NPO_4^-$ (m/z 180.04, to represent phosphatidylethanolamine (PE)), $C_{27}H_{43}O^-$ + $C_{27}H_{45}O^-$ (m/z 383.32 + m/z 385.37, to represent cholesterol) and $Fe(CN)_2^-$ (m/z 107.94, to represent heme[50]).

Histological analyses

Primary antibodies and conditions used for immunohistochemistry and immunofluorescence are listed in Table III in the Supplement. For immunohistochemistry, acetone-fixed

cryosections were incubated overnight at 4°C with primary antibodies. Incubation with biotinylated secondary antibodies was followed by detection with ABC peroxidase kit, using Warp Red (Biocare Medical) as color substrates and hematoxylin counterstaining (Vector Laboratories).

For immunofluorescent stainings, acetone-fixed sections of aortic valves were incubated overnight at 4°C with primary antibodies. Fluorescence emitted by secondary antibodies and by DAPI was captured with a confocal microscope (Leica TCS SP5).

Valvular interstitial cells (VICs) Isolation and stimulation

Human aortic valves were immersed in cell culture medium (DMEM, 10% fetal calf serum, 100 units/ml penicillin, 100 µg/ml streptomycin, 1 mM sodium pyruvate, 10 mM HEPES and 2 mM L-glutamine) (Gibco) immediately after aortic valve replacement surgery. Next, valves were digested with an enzymatic cocktail composed by collagenase I and dispase II for 16 h. VICs were seeded in tissue culture polystyrene containers (Corning) and medium was changed every second day. Cells were used between passages 2 and 4.

***In vitro* Calcification and RvE1 Stimulation**

VICs (6000 cells per well) were seeded in 96-well plates (Corning). Calcification was induced by culturing VICs for 9 days in cell culture medium (DMEM, 5% fetal calf serum, 100 units/ml penicillin, 100 µg/ml streptomycin, 1mM sodium pyruvate, 10 mM HEPES and 2 mM L-glutamine) supplemented with 2.6 mM inorganic phosphate (Sigma). RvE1 (100 nM) (Cayman Chemicals) or ethanol (0.01%) were added to the calcification medium, and changed every fother day. Calcification was assessed by Osteoimage Mineralization Assay (Lonza) according to manufacturer's protocol and validated by Alizarin Red staining.

Mouse echocardiography

Mice were anesthetized by 2.5% Isoflurane for mice that only went through echocardiography and by ketamine/xylazine (100 mg/kg and 16 mg/kg, respectively) i.p. in mice going through surgical intervention and echocardiography. After sleep induction, hair was removed with a hair removal cream in order to optimize the acoustic interface. Left ventricular (LV) dimensions were measured in parasternal-long axis in M-Mode (Visualsonics-Vevo 2100). Ejection fraction (EF) was calculated as (End diastolic volume (EDV) – End systolic volume (ESV))/EDV. Pulse-wave Doppler mode was used with a 55° angle correction in parasternal-long axis and right-suprasternal-long axis view for acquisitions of transvalvular velocities. Color Doppler was used to determine presence of regurgitation. M-Mode was used for cusp-separation evaluation in left-parasternal-long axis view using the inner-edge to inner-edge convention to assess distance. Left ventricular mass (LV mass) was calculated using the Devereux formula as previously described[51]. Echocardiographic measurements were performed in a blinded manner.

Aortic valve wire injury

ChemR23$^{+/+}$ (C57BL/6J) and ChemR23$^{-/-}$ mice were fed in a chow diet and until 10 weeks of age. Then, two weeks before surgery, mice were fed in a safflower oil rich diet (10% v/w) and maintained in this diet until sacrifice. Mice were operated according to the protocol established by Honda *et al.* for aortic valve injury[21]. In brief, animals were anesthetized with ketamine/xylazine (100 mg/kg and 16 mg/kg, respectively) i.p. After sleep induction the common right carotid artery was exposed, and the blood flow was stopped with a knot ligation on the proximal and distal part. A small incision was made to the exposed arterial segment

and a spring guide wire (0.36 mm of diameter) was inserted. The proximal knot was carefully opened, and the wire was guided to the ascending aorta, where it was identified through echocardiography under the parasternal-long axis view in B mode. The wire was carefully inserted into the left ventricle, passing the aortic valve. Aortic valve leaflet scratching was performed by means of moving the wire in and out of the left ventricle 20 times, and rotating the wire 50 times when it was placed inside left ventricle. After valve injury, the wire was carefully removed and a permanent carotid ligation was performed to avoid hemorrhage. Finally, the skin wound was closed with sutures and mice were monitored until awakening. Buprenorphine (0.1 mg/kg) was administered as analgesia by subcutaneous injection during 3 consecutive days starting right after surgery.

Tissue collection and analysis

Animals were put to death with CO_2. Blood was collected by cardiac puncture into EDTA-coated tubes and vascular perfusion was performed with 10 ml sterile RNase-free phosphate buffered saline (PBS). Blood cell counts were analyzed using an automated hematology analyzer (Scil Vet abc hemocounter). Plasma cholesterol and triglycerides were measured using enzymatic colorimetric kits (Randox Lab). Heart apex was snap-frozen for n-3 PUFA analysis and aortic roots were preserved in OCT for valve analysis followed by serial sectioning on a cryostat.

Lipoprotein profile

Plasma was fractionated using a Superose 6 10/300 GL column (GE Healthcare) coupled to a Prominence UFLC system (Shimadzu) and equilibrated with Tris-buffered saline at pH 7.4. Next, fractions of 200 µl were collected with a Foxy Jr fraction collector (Teldyne Isco) for

subsequent detection of cholesterol and triglycerides with the enzymatic kits mentioned above.

Evaluation of aortic valve leaflet area and thickness

Aortic valve morphology, in terms of leaflet area and leaflet thickness [19] were measured as previously described[52]. In brief, 5-7 sections covering the aortic valve were collected every 100 µm throughout the aortic root. Care was taken to establish the first level perpendicular to the insertion of the three aortic valve cusps, as previously described[52]. At each level, the average of the aortic valve leaflet area and thickness of the 3 leaflets was calculated. The level with highest aortic valve leaflet area was retained in each mouse for correlations. Quantification was performed using the software NDPview 2.

Evaluation of aortic valve leaflet calcification

Calcification was assessed by Alizarin Red staining as previously described[11]. In brief, 2% Alizarin Red was diluted in dH_2O, filtered, and pH adjusted to 4.1-4.3 with either 10% ammonium hydroxide or HCl. For histochemistry, formaldehyde fixed slides were hydrated, incubated for 2 minutes in 2% Alizarin Red, dehydrated in acetone, followed by acetone-xylene (1/1 v/v) solution, and cleared in xylene. Slides were mounted with a synthetic mounting medium (PERTEX, Histolab). Calcification area was assessed by measuring 5 sections collected every 100 µm of the aortic root. Calcification by Alizarin Red staining, instead Von Kossa staining, was chosen because the Von Kossa black staining can be confused with the presence of black-brown melanin deposits in the aortic leaflets[53]. Quantification was performed using the software ImageJ 1.48v.

Supplemental figures and tables

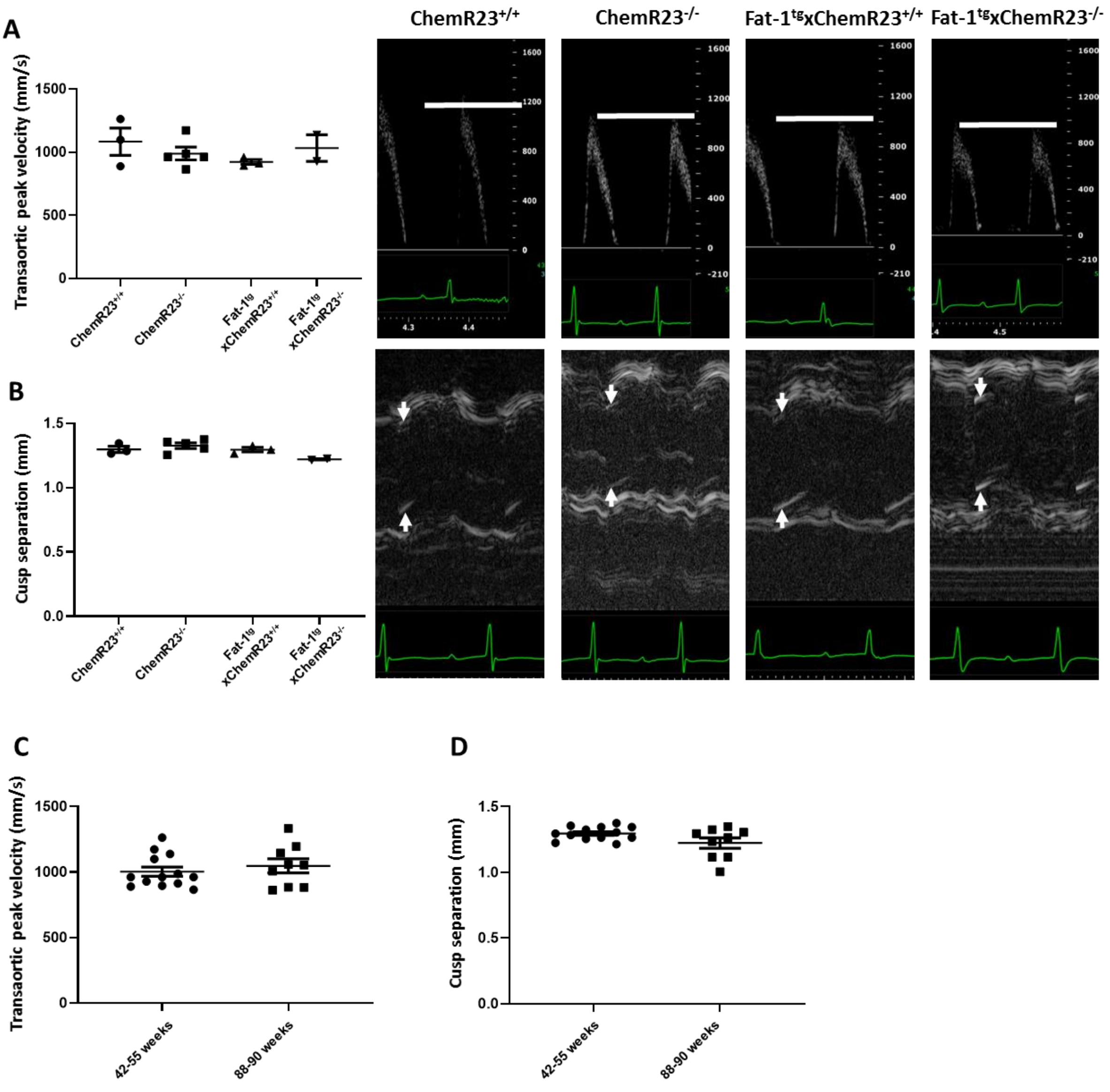

Supplemental Figure I. Control mice do not develop increased Vmax and reduced aortic valve cusp separation. (A) Vmax and **(B)** Cusp separation at 42-55 weeks old ChemR23$^{+/+}$ (n=3), ChemR23$^{-/-}$ (n=5), Fat-1^{tg}xChemR23$^{+/+}$ (n=3), and Fat-1^{tg}xChemR23$^{-/-}$ (n=2) mice. **(C)** Vmax and **(D)** Cusp separation comparing 42-55 weeks old (n=13) and 88-90 weeks old (n=9) mice. Representative Doppler and M-Mode tracings are shown at 42-55 weeks old. Data are

presented as individual values with horizontal lines representing mean±SEM. Statistical significances were evaluated using either a 1- way repeated measures ANOVA followed by Holm-Sidak multiple comparison test.

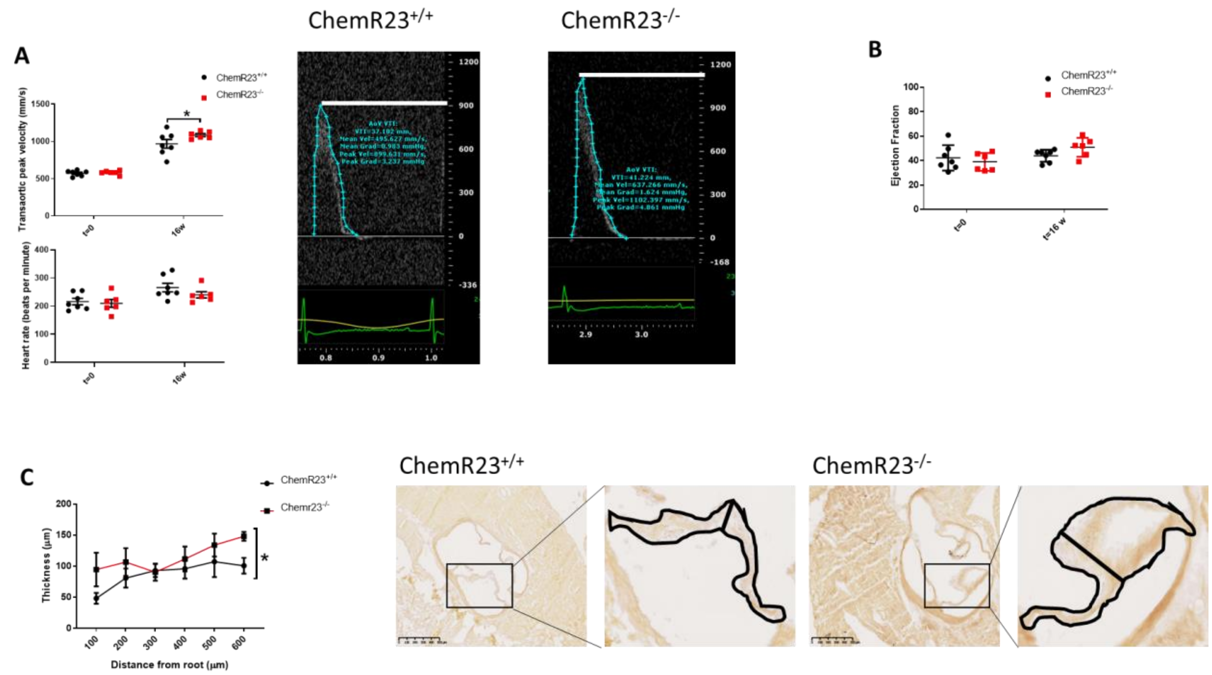

Supplemental Figure II. Targeted deletion of ChemR23 induces higher Vmax and increased thickening of the aortic valve after aortic valve wire injury. (A) Vmax before (t=0) and after (t=16w) aortic valve wire injury in ChemR23$^{+/+}$ (n=8) and ChemR23$^{-/-}$ (n=6) mice (top), and heart rate before (t=0) and after (t=16w) aortic valve wire injury (bottom). **(B)** Ejection fraction at t=0 and t16w. **(C)** Aortic valve leaflet thickness. Data are presented as either individual values with horizontal lines representing mean±SEM. Statistical significances were evaluated using a 2-way ANOVA followed by Holm-Sidak multiple comparison test. *P<0.05.

Supplemental Table I. Patient characteristics stratified according to HS-Omega-3 index above and below median.

	High HS-Omega-3 index (n=16)	Low HS-Omega-3 index (n=16)	*p*
**Age (years)**	73.9±1.4	72.3±1.44	0.45
**Sex (% male, n)**	62.5 (10)	75 (12)	0.70
**Smoking (% never, n)**	56.3 (9)	43.8 (7)	1
**BMI**	28.6±1.43	28.0±0.93	0.74
	**COMORBIDITIES**		
**Diabetes (% yes, n)**	18.8 (3)	25 (4)	1
**Hypertension (% yes, n)**	75 (12)	75 (12)	1
**Renal disease (% yes, n)**	6.3 (1)	6.3 (1)	1
**CAD (% yes, n)**	43.8 (7)	56.3 (9)	0.72
**PAD/stroke (% yes, n)**	18.8 (3)	6.3 (1)	0.6
**OSAS (% yes, n)**	0 (0)	18.8 (3)	0.23
	**TREATMENTS**		
**ASA (% yes, n)**	50 (8)	43.8 (7)	1
**Beta blockers (% yes, n)**	56.3 (9)	43.8 (7)	0.72
**ACEi/ARB (% yes, n)**	68.8 (11)	68.8 (11)	1
**Calcium blockers (% yes, n)**	25 (4)	31.3 (5)	1
**Diuretics (% yes, n)**	56.3 (9)	56.3 (9)	1
**Lipid lowering (% yes, n)**	62.5 (10)	50 (8)	0.72
**DOAC/Warfarin (% yes, n)**	25 (4)	31.3 (5)	1
	**LABORATORY PARMETERS**		
**Creatinine (µmol/l)**	86.8± 5.7	95.8±6.03	0.29
**Calcium (mmol/l)**	2.4±0.04	2.4±0.02	0.27
**HbA1c (mmol/mol)**	40.5±1.16	39.5±1.81	0.63
**Cystatin C (mg/l)**	1.1±0.08	1.2±0.1	0.47
**CRP>1 µg/ml (% yes, n)**	50 (8)	62.5 (10)	0.72
**eGFR>60 ml/min/1.73 m^2 (% yes, n)**	62.5 (10)	50 (8)	0.72
	**ECHOCARDIOGRAPHIC PARAMETERS**		
**EF>55% (% yes, n)**	75 (9)	72 (8)	1
**Vmax (m/s)**	4.4±0.13	4.4±0.15	0.67
**Mean gradient (mmHg)**	45.9±2.43	49.3±3.24	0.41
**Dimensionless index**	0.2±0.01	0.2±0.01	0.37

Data are presented as mean±SEM; Student *t*-test was used for continuous variables and Fisher's exact test for categorical variables).

Abbreviations: body mass index (BMI), coronary artery disease (CAD), peripheral artery disease (PAD), obstructive sleep apnea syndrome (OSAS), acetyl salicylic acid (ASA), angiotensin converting enzyme inhibitors/angiotensin II receptor blockers (ACEi/ARB), direct oral anticoagulants (DOAC), glycated hemoglobin (HbA1c), C reactive protein (CRP), estimated glomerular filtration rate (eGFR), ejection fraction (EF), maximum velocity (Vmax).

Supplemental Table II. GSEA analysis showing most differentially expressed KEGG pathways comparing high and low HS-Omega-3 Index in non-calcified and calcified human valve tissue.

Non-calcified valve tissue			Calcified valve tissue		
Pathway	p	q	Pathway	p	q
CELL CYCLE	0	0	LINOLEIC ACID METABOLISM	0	0
P53 SIGNALING PATHWAY	0	0	NOD LIKE RECEPTOR SIGNALING PATHWAY	0	0
ARACHIDONIC ACID METABOLISM	0	0.008	CELL CYCLE	0	0
GLYCOSYLPHOSPHATIDYLINOSITOL GPI ANCHOR BIOSYNTHESIS	0.006	0.011	APOPTOSIS	0	0.004
ECM RECEPTOR INTERACTION	0.004	0.026	ARACHIDONIC ACID METABOLISM	0	0.006
LYSINE DEGRADATION	0	0.031	CYSTEINE AND METHIONINE METABOLISM	0	0.017
ARGININE AND PROLINE METABOLISM	0.010	0.086	CHEMOKINE SIGNALING PATHWAY	0	0.021
WNT SIGNALING PATHWAY	0	0.089	PROPANOATE METABOLISM	0.002	0.025
RETINOL METABOLISM	0.003	0.091	RIG I LIKE RECEPTOR SIGNALING PATHWAY	0	0.030
GLYCOSAMINOGLYCAN BIOSYNTHESIS CHONDROITIN SULFATE	0.017	0.094	PURINE METABOLISM	0	0.034
LINOLEIC ACID METABOLISM	0.013	0.110	PYRIMIDINE METABOLISM	0	0.047
RIBOFLAVIN METABOLISM	0.023	0.105	N GLYCAN BIOSYNTHESIS	0.004	0.052
BIOSYNTHESIS OF UNSATURATED FATTY ACIDS	0.038	0.106	P53 SIGNALING PATHWAY	0.008	0.067
CYTOKINE CYTOKINE RECEPTOR INTERACTION	0	0.106	TOLL LIKE RECEPTOR SIGNALING PATHWAY	0.005	0.069
TGF BETA SIGNALING PATHWAY	0.004	0116	CITRATE CYCLE TCA CYCLE	0.028	0.092
RENIN ANGIOTENSIN SYSTEM	0.024	0.117	PHOSPHATIDYLINOSITOL SIGNALING SYSTEM	0.008	0.103
FOCAL ADHESION	0	0.120	VALINE LEUCINE AND ISOLEUCINE DEGRADATION	0.019	0.103
CYSTEINE AND METHIONINE METABOLISM	0.030	0.122	MTOR SIGNALING PATHWAY	0.009	0.114
TERPENOID BACKBONE BIOSYNTHESIS	0.068	0.138	RETINOL METABOLISM	0.002	0.114
OTHER GLYCAN DEGRADATION	0.031	0.145	PHENYLALANINE METABOLISM	0.035	0.116

Supplemental Table III. Antibodies used for immunohistochemistry.

**Primary antibody**	**Host**	**Source**	**Dilution**	**Use**
Anti-CD68	Rat	MCA1957 (Serotec)	1:20000	IHC
Anti-CD206	Rat	MCA2235 (BioRad)	1:50	IHC
Anti-Arg1	Goat	ab60176 (Abcam)	1:50	IHC
Anti-iNOS	Rabbit	ab15323 (Abcam)	1:100	IHC
Anti-ChemR23	Rabbit	ab150491 (Abcam)	1:100	IHC/IF
Anti-SMA	Mouse	M0851 (Dako)	1:100	IF
Anti-Vimentin	Mouse	M0725 (Dako)	1:100	IF
**Secondary antibody**	**Host**	**Source**	**Dilution**	**Use**
Biotinylated Anti-Rabbit	Goat	BA-1000 (Vector Labs)	1:200	IHC
Biotinylated Anti-Goat	Horse	BA-9500 (Vector Labs)	1:200	IHC
Biotinylated Anti-Rat	Rabbit	BA-4001 (Vector Labs)	1:200	IHC
Anti-Rabbit	Goat	DI-1594 (Vector Labs)	1:300	IF
Anti-Mouse	Horse	DI-2488 (Vector Labs)	1:300	IF
**Controls**	**Host**	**Source**	**Dilution**	**Use**
Anti-Rat IgG2a; Isotype	Rat	MAB006 (R&D)	1:100	IHC

Abbreviations: Immunohistochemistry (IHC), immunofluorescence (IF), cluster of differentiation (CD), arginase 1 (Arg1), inducible nitric oxide synthase (iNOS), smooth muscle actin (SMA).

Supplemental Table IV. Gas chromatography analyses in ventricular myocardium of $Apoe^{-/-}$ $xChemR23^{+/+}$ and $Fat\text{-}1^{tg}xApoe^{-/-}xChemR23^{+/+}$.

	$Apoe^{-/-}$ $xChemR23^{+/+}$ (n=6)	$Fat\text{-}1^{tg}xApoe^{-/-}$ $xChemR23^{+/+}$ (n=6)	*T-test*	*Holm-Sidak*
**Fatty acid**	**Mean±SEM**	**Mean±SEM**	**p**	**Adjusted p**
**C14:0**	0.14±0.007	0.16±0.008	0,198	N.S
**C16:0**	12.96±0.04	14.18±0.24	0,0006	**0.01**
**C16:1n7t**	0.06±0.003	0.07±0.002	0,006	N.S
**C16:1n7**	0.69±0.05	0.65±0.04	0,538	N.S
**C18:0**	16.22±0.26	14.95±0.22	0,004	N.S
**C18:1t**	0.26±0.03	0.21±0.02	0,232	N.S
**C18:1n9**	11.52±0.60	11.07±0.70	0,634	N.S
**C18:2n6tt**	0.01±0.00	0.01±0.00	N/A	N/A
**C18:2n6ct**	0.00±0.00	0.00±0.00	N/A	N/A
**C18:2n6tc**	0.02±0.002	0.03±0.002	0,599	N.S
**C18:2n6**	19.23±0.57	21.94±0.69	0,012	N.S
**C20:0**	0.16±0.01	0.17±0.01	0,234	N.S
**C18:3n6**	0.03±0.002	0.04±0.004	0,017	N.S
**C20:1n9**	0.48±0.04	0.59±0.03	0,033	N.S
**C18:3n3**	0.08±0.006	0.16±0.02	0,004	N.S
**C20:2n6**	0.19±0.02	0.23±0.01	0,157	N.S
**C22:0**	0.22±0.009	0.26±0.02	0,080	N.S
**C20:3n6**	0.89±0.07	0.77±0.03	0,121	N.S
**C20:4n6**	11.97±0.30	4.29±0.30	<0,000001	**<0.000001**
**C24:0**	0.04±0.007	0.04±0.005	0,441	N.S
**C20:5n3**	0.05±0.004	0.35±0.02	<0,000001	**0.000002**
**C24:1n9**	0.05±0.005	0.05±0.004	0,451	N.S
**C22:4n6**	0.75±0.03	0.14±0.02	<0,000001	**<0.000001**
**C22:5n6**	2.65±0.26	0.21±0.04	0,000003	**0.00006**
**C22:5n3**	1.17±0.13	2.24±0.05	0,00001	**0.0003**
**C22:6n3**	20.19±0.46	27.21±0.29	<0,000001	**0.000003**
**HS-Omega-3 Index**	20.23±0.46	27.56±0.29	<0,000001	**0.000002**

Data are presented as mean±SEM. Statistical significances were evaluated using a multiple t-test followed by a Holm-Sidak multiple comparison correction. N.S: p>0.05.

Abbreviations: non-significant (N.S), non-applicable (N/A)

Supplemental Table V. Full echocardiography profile in 72 weeks old Apoe$^{-/-}$xChemR23$^{+/+}$, Apoe$^{-/-}$xChemR23$^{-/-}$, Fat-1^{tg}xApoe$^{-/-}$xChemR23$^{+/+}$, and Fat-1^{tg}xApoe$^{-/-}$xChemR23$^{-/-}$ mice.

	**Apoe$^{-/-}$ xChemR23$^{+/+}$ (n=12)**	**Apoe$^{-/-}$ xChemR23$^{-/-}$ (n=13)**	**Fat-1^{tg}xApoe$^{-/-}$ xChemR23$^{+/+}$ (n=6)**	**Fat-1^{tg}xApoe$^{-/-}$ xChemR23$^{-/-}$ (n=13)**	***(One-Way ANOVA)***
**PARAMETER**	Mean±SEM	Mean±SEM	Mean±SEM	Mean±SEM	*p*
**VTI** (mm)	76.65±2.96	94.64±4.65	55.05±6.43	90.11±4.61	**<0.0001**
**Dimensionless Index**	0.50±0.02	0.40±0.02	0.70±0.06	0.44±0.02	**<0.0001**
**Mean Gradient** (mmHg)	4.35±0.32	6.47±0.49	2.61±0.49	5.49±0.47	**<0.0001**
**Peak Velocity** (m/s)	1.76±0.06	2.16±0.09	1.33±0.12	1.98±0.09	**<0.0001**
**Cusp Separation** (mm)	0.94±0.04	0.74±0.04	1.17±0.07	0.86±0.07	**0.0003**
**LVIDd** (mm)	4.20±0.08	4.16±0.05	4.05±0.09	4.09±0.09	N.S
**LVIDs** (mm)	3.1±0.10	3.10±0.07	3.04±0.11	3.07±0.08	N.S
**EF** (%)	51.88±2.33	50.37±1.87	49.70±3.05	50.02±1.60	N.S
**FS**(%)	26.49±1.50	25.45±1.17	25±1.85	25.16±0.98	N.S
**IVSd** (mm)	0.88±0.07	1.15±0.04	0.85±0.1	0.97±0.07	**0.04**
**LVPWd** (mm)	0.84±0.03	0.82±0.04	0.79±0.06	0.85±0.05	N.S
**LV mass** (mg)	160±9.20	168.2±7.80	124.5±8.57	146.5±9.50	**0.03**
**HR** (bpm)	383.3±13.79	385.3±13.39	404.5±19.03	377.8±13.57	N.S

Data are presented as mean±SEM. Statistical significances were evaluated using a 1-way ANOVA. N.S: p>0.05.

Abbreviations: velocity time integral (VTI), left ventricular inner diameter in diastole (LVIDd), left ventricular inner diameter in systole (LVIDs), ejection fraction (EF), fractional shortening (FS), interventricular septum in diastole (IVSd), left ventricular posterior wall in diastole (LVPWd), heart rate (HR), beats per minute (bpm), left ventricular mass (LV mass), non-significant (N.S).

Supplemental Table VI. Heart rates (HR) at echocardiography at 52 and 64 weeks old $Apoe^{-/-}xChemR23^{+/+}$, $Apoe^{-/-}xChemR23^{-/-}$, $Fat\text{-}1^{tg}xApoe^{-/-}xChemR23^{+/+}$, and $Fat\text{-}1^{tg}xApoe^{-/-}xChemR23^{-/-}$ mice.

	**$Apoe^{-/-}$ $xChemR23^{+/+}$ (n=12)**	**$Apoe^{-/-}$ $xChemR23^{-/-}$ (n=13)**	**$Fat\text{-}1^{tg}xApoe^{-/-}$ $xChemR23^{+/+}$ (n=6)**	**$Fat\text{-}1^{tg}xApoe^{-/-}$ $xChemR23^{-/-}$ (n=13)**	***(One-Way ANOVA)***
**Time Point**	Mean±SEM	Mean±SEM	Mean±SEM	Mean±SEM	*p*
**52 weeks** (bpm)	371.3±13.93	394.4±13.25	396.2±21.17	378.5±11.39	N.S
**64 weeks** (bpm)	387.4±9.344	383.2±18.77	402.3±14.29	383.5±10.36	N.S

Data are presented as mean±SEM. Statistical significances were evaluated using a 1-way ANOVA. N.S: $p>0.05$.

Abbreviations: beats per minute (bpm), non-significant (N.S).

Supplemental Table VII. Blood cell counts in 72 weeks old Apoe$^{-/-}$xChemR23$^{+/+}$, Apoe$^{-/-}$xChemR23$^{-/-}$, Fat-1^{tg}xApoe$^{-/-}$xChemR23$^{+/+}$, and Fat-1^{tg}xApoe$^{-/-}$xChemR23$^{-/-}$ mice.

	**Apoe$^{-/-}$ xChemR23$^{+/+}$ (n=9)**	**Apoe$^{-/-}$ xChemR23$^{-/-}$ (n=12)**	**Fat-1^{tg}xApoe$^{-/-}$ xChemR23$^{+/+}$ (n=4)**	**Fat-1^{tg}xApoe$^{-/-}$ xChemR23$^{-/-}$ (n=13)**	***(One-Way ANOVA)***
**PARAMETER**	**Mean±SEM**	**Mean±SEM**	**Mean±SEM**	**Mean±SEM**	***p***
**WBC**	5.8±0.58	6.28±0.53	6.58±0.28	8.3±0.90	N.S
**PLT**	1551±358.6	1532±323.8	1583±36	1518±132.5	N.S
**%Lym**	72.29±3.17	73.08±2.1	63.5±4.34	71.58±2.02	N.S
**%Mon**	6.63±0.40	6.18±0.42	6.8±0.19	6.84±0.37	N.S
**%Gra**	21.08±2.94	20.73±1.77	29.7±4.25	21.58±1.78	N.S
**#Lym**	4.13±0.47	4.57±0.44	4.13±0.36	5.91±0.68	N.S
**#Mon**	0.33±0.04	0.34±0.03	0.4±0.040	0.52±0.08	N.S
**#Gra**	1.33±0.23	1.38±0.14	2.05±0.26	1.87±0.22	N.S

Data are presented as mean±SEM. Statistical significances were evaluated using a 1-way ANOVA. N.S: $p>0.05$.

Abbreviations: white blood cells (WBC), platelets (PLT), percentage of lymphocytes (%Lym), percentage of monocytes (%Mon), percentage of granulocytes (%Gra), number of lymphocytes (#Lym), number of monocytes (#Mon), and number of granulocytes (#Gra), non-significant (N.S).

Supplemental Table VIII. ChemR23 correlations with M2 macrophage markers in human aortic valves.

**Non-Calcified valve tissue**						
	CD206	***ARG1***	***CD163***	***CD209***	***HMOX1***	***CD200R1***
**Rho**	0.455	-0.0216	0.494	0.518	0.443	0.207
p	**0.000182**	0.865	**0.0000386**	**0.0000141**	**0.000272**	0.1
**Calcified valve tissue**						
	CD206	***ARG1***	***CD163***	***CD209***	***HMOX1***	***CD200R1***
**Rho**	0.506	0.0988	0.474	0.664	0.409	0.246
p	**0.0000242**	0.436	**0.0000868**	**0.0000002**	**0.00085**	**0.0499**

Abbreviations: cluster of differentiation (CD), arginase 1 (ARG1), heme oxygenase 1 (HMOX1).

Supplemental Table IX. Blood cell counts in ChemR23$^{+/+}$ and ChemR23$^{-/-}$.

	**ChemR23$^{+/+}$ (n=5)**	**ChemR23$^{-/-}$ (n=6)**	***T-Test***
**PARAMETER**	**Mean±SEM**	**Mean±SEM**	***p***
**WBC**	4.48±0.49	5.63±1.34	N.S
**PLT**	494±32.9	463.2±88.7	N.S
**%Lym**	73.42±1.99	70.7±2.70	N.S
**%Mon**	6.96±1.43	6.47±0.83	N.S
**%Gra**	20.3±1.71	23.17±2.57	N.S
**#Lym**	3.26±0.38	3.98±0.97	N.S
**#Mon**	0.26±0.09	0.35±0.12	N.S
**#Gra**	1.02±0.11	1.3±0.30	N.S

Data are presented as mean±SEM. Statistical significances were evaluated using a Student *t*-test. N.S: $p>0.05$.

Abbreviations: white blood cells (WBC), platelets (PLT), percentage of lymphocytes (%Lym), percentage of monocytes (%Mon), percentage of granulocytes (%Gra), number of lymphocytes (#Lym), number of monocytes (#Mon), and number of granulocytes (#Gra), non-significant (N.S).
